# 3D Printable Filaments Made of Biobased Polyethylene Biocomposites

**DOI:** 10.3390/polym10030314

**Published:** 2018-03-13

**Authors:** Daniel Filgueira, Solveig Holmen, Johnny K. Melbø, Diego Moldes, Andreas T. Echtermeyer, Gary Chinga-Carrasco

**Affiliations:** 1 Department of Chemical Engineering, Edificio Isaac Newton, Lagoas-Marcosende s/n, University of Vigo, 36310 Vigo, Spain; danmartinez@uvigo.es (D.F.); diego@uvigo.es (D.M.); 2 Department of Mechanical and Industrial Engineering, NTNU, 7491 Trondheim, Norway; solveig.holmen91@gmail.com (S.H.); andreas.echtermeyer@ntnu.no (A.T.E.); 3 RISE PFI, Høgskoleringen 6b, 7491 Trondheim, Norway; johnny.melbo@rise-pfi.no

**Keywords:** laccase, grafting, TMP, BioPE, biocomposites, lauryl gallate, octyl gallate, 3D printing

## Abstract

Two different series of biobased polyethylene (BioPE) were used for the manufacturing of biocomposites, complemented with thermomechanical pulp (TMP) fibers. The intrinsic hydrophilic character of the TMP fibers was previously modified by grafting hydrophobic compounds (octyl gallate and lauryl gallate) by means of an enzymatic-assisted treatment. BioPE with low melt flow index (MFI) yielded filaments with low void fraction and relatively low thickness variation. The water absorption of the biocomposites was remarkably improved when the enzymatically-hydrophobized TMP fibers were used. Importantly, the 3D printing of BioPE was improved by adding 10% and 20% TMP fibers to the composition. Thus, 3D printable biocomposites with low water uptake can be manufactured by using fully biobased materials and environmentally-friendly processes.

## 1. Introduction

Biocomposites are expected to contribute to the production of environmentally sound products [1]. Materials are classified as biocomposites if at least one of the constituents is derived from biological material [2]. Most biocomposites used today are made of a synthetic polymer reinforced with lignocellulosic fibers. Such fibers are low density biodegradable materials with low cost, high availability worldwide, and acceptable specific strength properties [3,4].

Lignocellulosic fibers need to be processed at low temperatures since their degradation is initiated at about 200 °C [5,6,7]. This characteristic limits the use of several polymers, such as polyethylene teraphtalate (PET), as matrix phase in the manufacturing of biocomposites, since their melting temperature exceeds the fiber´s degradation temperature. A suitable polymer is polyethylene (PE), a thermoplastic polymer with a processing temperature low enough to avoid the degradation of the lignocellulosic fibers [8]. Hence, PE is one of the most used polymers in the manufacturing of biocomposites [3]. Traditionally, the PE used is an oil-derived polymer with a carbon footprint higher than biopolymers like polylactic acid [9]—not the best choice from an environmental perspective. However, BioPE is available and industrially manufactured from materials, such as sugarcane, sugar beet, or wheat grains. It is worth mentioning that plants are a renewable feedstock, which consume CO_2_ in each annual growth cycle. Hence, the manufacturing of 1 Ton of biobased PE (BioPE) from sugarcane could capture 2.5 Tons of CO_2_ from the atmosphere, whenever solar energy is used for energy production [10]. Therefore, BioPE clearly contributes to reducing the carbon footprint, compared to fossil PE. On the other hand, BioPE exhibits the same physical and chemical characteristics as fossil-based PE which allows its direct implementation in industrial manufacturing processes [10].

Regarding the properties of biocomposites, the physical characteristics of the fibers such as fiber length and aspect ratio have a dramatic impact on the mechanical properties of the biocomposites [11,12,13]. This effect is already well known from traditional composites [14]. Thermomechanical pulp (TMP) fibers are a relatively cheap raw material with a higher aspect ratio than other biobased materials (e.g., wood flour), leading to the manufacturing of biocomposites with good mechanical properties [15,16]. One challenge of using TMP fibers in a PE matrix is that the fibers are highly hydrophilic [17] (like all natural fibers) and the matrix is hydrophobic. The fibers and matrix are chemically incompatible resulting in poor interfacial adhesion. The incompatibility also leads to clustering of the fibers, preventing good dispersion and possibly formation of voids. A further problem is swelling of the TMP-reinforced biocomposites due to absorption of water by the fibers from the environment, which can affect the dimensional stability of the biocomposite products.

Surface modification of lignocellulosic fibers is a promising strategy to improve the interfacial adhesion of the fiber-matrix system and dispersion of the fibers. Different compatibilizers, but also chemical and physical treatments have been proposed for the surface modification of the lignocellulosic fibers with interesting results [3,18]. However, the problem with the existing solutions is their environmental footprint. Treatments may be based on chemicals that are not ideal from an environmental point of view. Environmentally friendly alternatives are enzymatic treatments such as laccase-mediated reactions. Laccase is a phenoloxidase enzyme that has the capability to oxidize phenolic compounds leading to the formation of their corresponding phenoxy radicals which could be grafted onto the surface of the fibers, modifying their bio-chemical properties [19]. Although laccase cannot oxidize high-redox potential compounds, the addition of a mediator to the laccase-assisted treatment enables the oxidation of other chemical structures beyond phenolic compounds. Therefore, in the so-called laccase-mediator system (LMS), the oxidized mediators may oxidize chemical compounds such as sterols or fatty alcohols, which could not be oxidized by laccase itself [20,21]. In addition, the mediators could penetrate in regions of the lignocellulosic fibers which laccase cannot access due to steric hindrance. Previous studies have shown that TMP fibers could be modified by means of laccase-assisted reactions for the manufacturing of biocomposites [22] and particleboards [23,24], or for removing lipophilic extractives [25]. Additionally, biocomposites with low water uptake are expected to be an interesting material, for example, for injection molding of automotive parts, where the dimensional stability is a critical characteristic.

Most of the applications of the biocomposites reinforced with lignocellulosic fibers are related to the construction and automotive industries [26]. Nonetheless, new manufacturing processes are required in order to increase the applications of biocomposites and provide added-value to the lignocellulosic fibers. 3D printing offers a new perspective in the manufacturing of biocomposite products, which could be used in very specific applications such as medical prosthesis, regenerative medicine, or drug delivery [27,28,29,30]. Its possibilities of rapid prototyping and direct part fabrication make 3D printing one of the most promising and time/cost-efficient production processes for the industry. Moreover, 3D printing is not only cost efficient, but also facilitates production of parts with complex geometry, repairs, and assembly. In fact, important industrial sectors (e.g., medical, aerospace) are already applying 3D printing technology [31,32,33].

In the present study, BioPE and TMP fibers are used for biocomposite manufacturing. In order to reduce the water uptake of TMP fibers and of the corresponding biocomposites, the grafting of hydrophobic compounds was carried out by means of an eco-friendly enzymatic process. Hence, the new biocomposites are expected to have improved compatibility between their components, but also lower water absorption and improved suitability for 3D printing.

## 2. Materials and Methods 

### 2.1. Materials

Two series of biobased polyethylene (BioPE) were kindly provided by Braskem (Sao Paulo, Brazil). They were both high-density polyethylene (HDPE) with different melt flow index (MFI), 20 g/10 min (BioPE1) and 4.5 g/10 min for (BioPE2). The density of BioPE was practically the same, 0.955 and 0.954 g/cm^3^ respectively for BioPE1 and BioPE2.

Spruce TMP fibers were kindly provided by Norske Skog Saugbrugs (Halden, Norway). The chemical composition of TMP was 48.2% cellulose, 25.6% hemicellulose, 26% lignin, and 0.2% extractives. The average fiber length of the collected TMP fibers was 1.5 mm and the diameter was 33 μm [22].

Laccase from *Myceliophthora termophila* (NS51003) was supplied by Novozymes (Bagsværd, Denmark). The activity of the enzyme was calculated by the 2,2′-azino-bis(3-ethylbenzothiazoline-6-sulphonic acid) (ABTS) oxidation assay. One unit of activity was defined as the amount of enzyme that oxidized 1 μmol of ABTS per minute at 25 °C and pH 7 (0.1 M phosphate buffer).

Compatibilizer Licocene maleic anhydride polyethylene (MAPE) 4351 Fine Grain was provided by Clariant (Basel, Switzerland). The compatibilizer has an acid value of 42–49 mg KOH/g, a density (23 °C) of 0.98–1.00 g/cm^3^, a drop point of 120–126 °C, and a viscosity between 200–500 mPa·s.

The remaining chemical reagents were purchased from Sigma-Aldrich (St. Louis, MO, USA) at reagent grade and used without further purification.

### 2.2. TMP Fibers Modification

TMP fibers (18 g of oven dried pulp (odp)) were suspended in a 2 L reactor with 1.8 L of phosphate buffer (0.1 M, pH 7) at 50 °C for 30 min. Laccase enzyme (175 U/g odp) and guaiacol (G) (10 mM) were added to the solution 30 min before adding 80 mL of an acetone solution containing Octyl Gallate (OG) or Lauryl Gallate (LG) (0.15 M). After 2 h under agitation, the fibers were dried at room temperature and then washed with distilled water/acetone solution (60:40%, *v*/*v*) for 1 h at 50 °C. Finally, the fibers were repeatedly washed with distilled water and dried at 50 °C for 12 h.

### 2.3. Extrusion of Biocomposite Filaments 

An overview of the series that were prepared is given in Table 1. In order to obtain a homogeneous blend, BioPE pellets and TMP fibers were ground in a Thomas Wiley Mini-Mill Cutting mill to mesh 10 and 30, respectively. The average fiber length of the milled TMP fibers was 0.4 mm and the diameter was 38 μm [22]. The milled BioPE and the fibers were oven dried (105 °C during 1 h) and the blending was performed at two different TMP fiber loads, 10% and 20% weight fraction. MAPE compatibilizer was added to the blends depending on the TMP fiber load; 1% and 2% MAPE for weight fraction 10% and 20% of fibers loads, respectively. The blend was extruded in a Noztek Xcalibur (Shoreham, UK), which has a single screw and three different heating chambers for the total control of the extrusion temperature.

Different temperatures were tested during the extrusion process of BioPE1 and BioPE2 with the TMP fibers. For temperatures lower than 150 °C, filaments with a high roughness and porosity were obtained due to a low melt flow of both BioPE1 and BioPE2. At the same time, for temperatures above 170 °C, significant foaming and deterioration of the fibers cell structure was observed. Hence, the best temperature conditions were found in the range of 150 and 165 °C. Such results were in accordance with Guo et al., who found that the critical temperature for the extrusion of HDPE/wood fibers composites was 170 °C [34]. Therefore, BioPE1 was extruded at 150, 155, and 160 °C and BioPE2 at 155, 160, and 165 °C, respectively for chambers 1, 2, and 3. The differences in the extrusion temperatures between BioPE1 and BioPE2 were due to the different MFI (20 and 4.5 g/10 min, respectively, for BioPE1 and BioPE2).

The speed of the screw extruder was 12 mm/s and the fan was set at 65%. Filaments with a diameter of approx. 2 mm were obtained. All the filaments were spooled with a Filabot spooler at the output of the extruder.

The Biobased polyethylene (BioPE) filaments were extruded once and twice to assess the evolution of filament’ thickness variation and porosity. Hence, the filaments obtained after the first extrusion were cut in small pellets (20 mm length), which were extruded again under the same conditions of the first extrusion.

### 2.4. Filament Morphology

Three random pieces of filaments, each 20 mm in length, were scanned in an Epson Perfection V750 (Long Beach, CA, USA) for quantification of thickness variation. The images were acquired in reflection and transmission modes with a resolution of 2400 dots per inch. The images acquired in transmission mode were automatically thresholded and binarized. The variation in thickness of the binarized filaments were quantified with a plugin for ImageJ developed for this purpose. The thickness variation is considered a measurement of the filament roughness and corresponds to the variation in thickness along each single filament piece (20 mm length, 3 replicates).

Pieces of filaments were used to estimate the void fraction of the filaments considering the weight of the filaments (*W*_i_, in g), the cross-sectional area (*A*_i_, in cm^2^) and length (*L*_i_, in cm) of the pieces, the estimated density of TMP fibers (1.56 × 10^−6^ g/cm^3^) and BioPE (0.955 g/cm^3^ for BioPE1 and 0.954 g/cm^3^ for BioPE2), and the mass fraction of fibers (*X*_F_) and BioPE (*X*_BioPE_) in the filaments. The void fraction was calculated as follows:

Void fraction (%) = ((Theoretical density − Real density)/Real density) × 100(1)

Real density = *W*_i_/(*A*_i_ × *L*_i_) (2)

Theoretical density = (*X*_F_ × 1.56 × 10^−6^) + (*X*_BioPE_ × *d*_BioPE_) (3)

It should be noted that the cross-sectional area *A*_i_ is obtained by measuring the diameter of the filaments and assuming a circular cross-section. This is a reasonably simple approach, but it considers the surface roughness as voids.

### 2.5. SEM Analysis

The filaments were embedded in epoxy resin and prepared for scanning electron microscopy (SEM), in backscatter electron imaging (BEI) mode [35]. The prepared samples were coated with carbon before visualization in BEI mode. Additionally, and for exemplification purposes, the surfaces of some fracture areas were visualized in secondary electron imaging (SEI) mode after coating the surface with a thin layer of gold. A Hitachi SU3500 Scanning Electron Microscope (Tokyo, Japan) was used for the analyses. The applied acceleration voltage and magnification were 5 kV and 50×, respectively. 

### 2.6. Water Uptake

Three test specimens (length = 60 ± 1 mm) of each filament were immersed in 40 mL of distilled water for 32 days. The samples were initially dried (50 °C for 24 h) and the dried weight (*W*_0_, in g) was measured. The samples were weighted every 24 h (*W*_i_). The water uptake of the filaments was measured by the following equation,

Water uptake (weight %) = ((*W*_i_ − *W*_0_)/*W*_0_) × 100 (4)

### 2.7. 3D Printing

BioPE filaments complemented with hydrophobized-TMP fibers were used for printing 3D model figures (Ø = 20 mm) in an Ultimaker Original 3D printer (Geldermalsen, The Netherlands). The diameter of the 3D printer nozzle was 0.4 mm and the print speed and temperature were set at 15 mm/s and 210 °C, respectively. The design of the model figures was performed with the ImageJ program (version 1.50i, National Institutes of Health, Bethesda, MD, USA).

## 3. Results and Discussion

### 3.1. Filaments Morphology and Porosity

**Figure 1:**
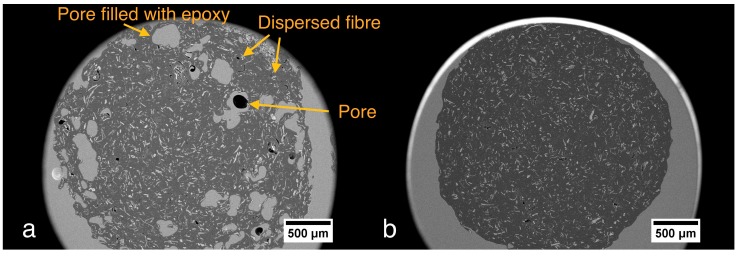
SEM cross-sectional image at 50× magnification. (**a**) B2-OGT20 after one extrusion; (**b**) B2-OGT20 after two extrusions.

All the manufactured filaments showed high roughness after the first extrusion. SEM images evidenced a heterogeneous distribution of the TMP fibers and relatively big pores in the polymer matrix (Figure 1a). Hence, a second extrusion under the same conditions was carried out to enhance the dispersion of the fibers in the BioPE matrix. As exemplified in Figure 1b, the fiber dispersion of the sample B2-20OGT was remarkably improved and relatively big pores were not detected after a second extrusion process.

**Figure 2:**
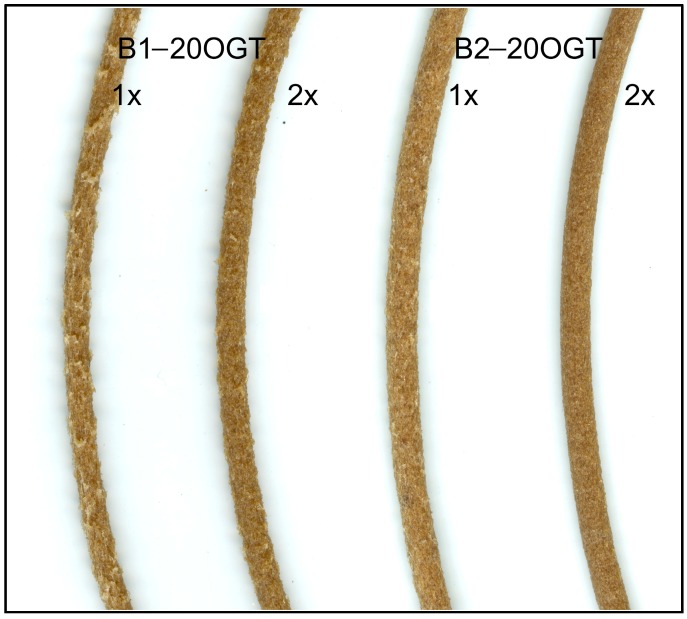
Representative filaments after first (1×) and second (2×) extrusion.

One parameter that could influence the reduction of the filaments’ porosity after the second extrusion could be the different shape of the feeding in the extruder. In the second extrusion, the feeding was added in the form of small pellets (20 mm in length; 2 mm in diameter) whereas in the first extrusion the feeding was added as powder of milled fibers and BioPE. Therefore, the pelletizing of the fibers and the BioPE before extruding could improve the blending in the extrusion chambers, leading to the manufacture of filaments with a lower porosity. Additionally, it is likely that the second extrusion process could reduce the fiber agglomerations. Therefore, the roughness and visual appearance of the filaments were significantly improved after the second extrusion (Figure 2).

**Figure 3:**
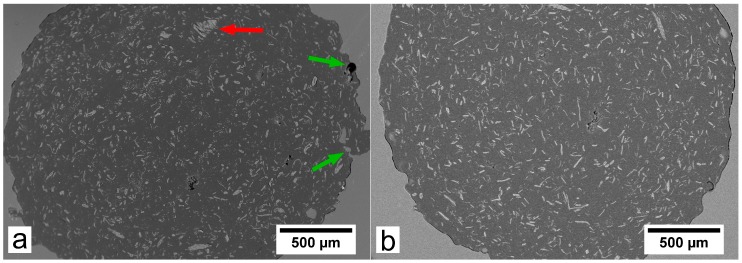
SEM cross-sectional image at 50× magnification. (**a**) B2-20T; (**b**) B2-20LGT. The red arrow indicates an agglomeration of fibers. The green arrows indicate surface pores probably caused by the relatively high surface roughness at this local area.

Regarding the different TMP fibers used to complement the BioPE matrix, it was observed that enzymatically LG-modified TMP fibers exhibited a more homogeneous distribution and apparently less fiber agglomeration in the BioPE2 matrix (Figure 3). A similar behaviour was observed with the OG-modified TMP fibers. Additionally, Figure 3a exemplifies some pores and cavities in the filament surface which may facilitate the water diffusion into the filament structure. The modified TMP fibers were hydrophobized by means of the enzymatic grafting of OG or LG. Such compounds possess an aliphatic chain, which apparently favored the chemical compatibility between the TMP fibers with the hydrophobic matrix.

**Figure 4:**
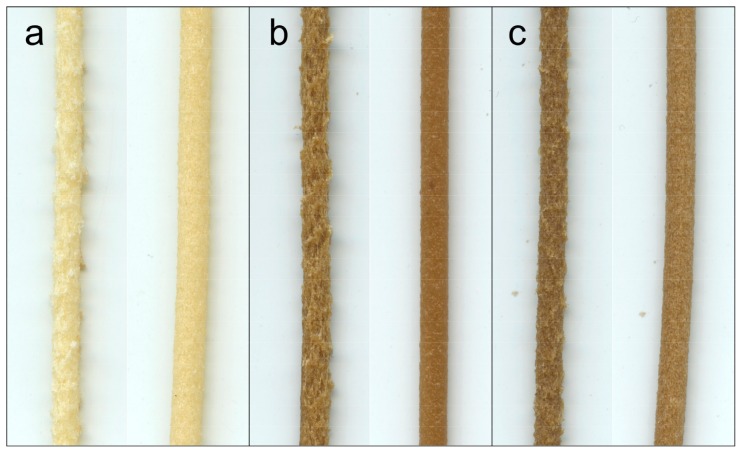
Comparison of the roughness in the filaments produced from BioPE1 (left) and BioPE2 (right) with fibers loads of 20%: (**a**) B1-T20 and B2-T20; (**b**) B1-LGT20 and B2-LGT20; (**c**) B1-OGT20 and B2-OGT20.

Nonetheless, the roughness of the filaments manufactured with BioPE1 was remarkably higher than those manufactured with BioPE2 (Figure 4), even after the second extrusion. The MFI of BioPE1 is 5-fold higher than the MFI of BioPE2, which means that BioPE1 has a lower viscosity and a higher capacity to flow than BioPE2. The difference in MFI is directly related to their average molecular weight. Although both biopolymers are HDPE, BioPE2 has a higher average molecular weight, which means that BioPE2 possesses an increased entanglement of chains and a less ordered structure than BioPE1 [36]. Hence, the higher proportion of entanglements of the BioPE2 could facilitate its blending with the TMP fibers during the extrusion process. On the other hand, due to the low viscosity, the speed at which the BioPE1 flows in the extruder could be remarkably higher than the flow of TMP fibers, probably leading to a heterogeneous distribution of the fibers along the filaments.

**Figure 5:**
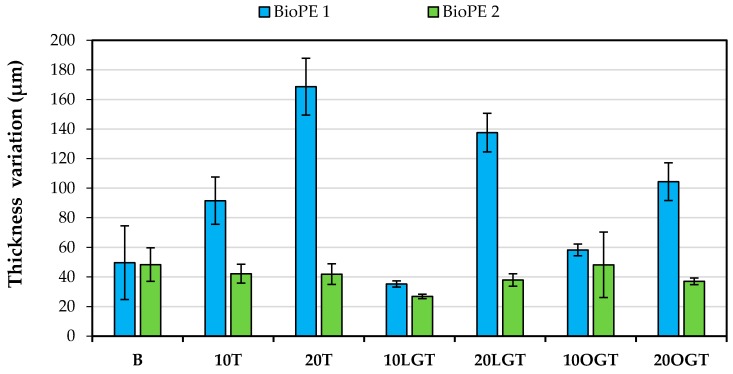
Thickness variation of the filaments as a function of the matrix (BioPE1 or BioPE2) and the thermomechanical pulp (TMP) fibers; BioPE (B), TMP fibers (T), LG-treated TMP fibers (LGT) and OG-treated TMP fibers (OGT).

Therefore, the thickness variation of the filaments was assessed in order to measure the differences in the filaments’ roughness. For all the manufactured filaments (diameter = 2 ± 0.1 mm), the thickness variation was lower for the BioPE2 series, compared to the corresponding BioPE1. Those differences were significantly larger for fiber loads of 20%, where the BioPE2-based filaments showed on average a 70% lower thickness variation respect to BioPE1-based filaments (Figure 5). Fewer differences between both matrices were found for fiber loads of 10% and especially with the use of enzymatically modified TMP fibers (10LGT and 10OGT). These results suggest that the lower MFI of BioPE2 enabled a better blending with the modified TMP fibers during the extrusion process, yielding relatively smooth and homogeneous filaments. On the contrary, the higher MFI of BioPE1 led to a poorer blending between the polymer matrix and the TMP fibers, thus yielding rough filaments with presumptively limited 3D printability.

**Figure 6:**
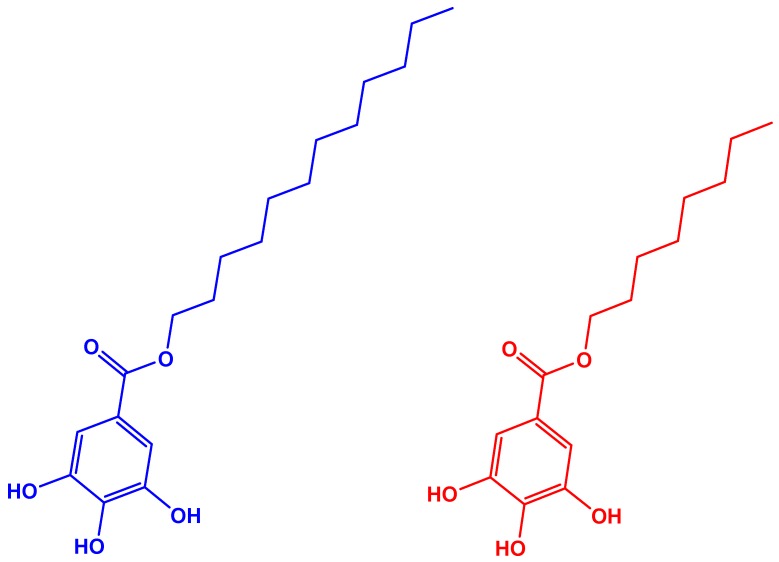
Chemical structure of Lauryl Gallate (LG) and Octyl Gallate (OG).

When the filaments were manufactured with hydrophobic fibers, their roughness was reduced in most cases. The lowest thickness variation was observed in the B2-10LGT series. The differences between B2-10LGT and B2-10OGT could be caused by the longer aliphatic chain of LG (Figure 6), which could significantly improve the chemical compatibility with the hydrophobic polymer matrix. However, previous work with both fibers (LGT and OGT) and polylactic acid (PLA) as polymeric matrix evidenced that OGT fibers had a better chemical compatibility with the PLA matrix [22]. These results suggest that the interfacial adhesion of the fiber-matrix system depends on both the degree of hydrophobicity of the TMP fibers, but also the chemical structure of the polymeric matrix.

The void fraction of the filaments is a measure of the filaments’ porosity. Hence, the density of the filaments was estimated and compared with the theoretical density of the filaments (Table 2). As expected, the use of TMP fibers produces a variable void volume depending on the matrix and fiber used, but mainly depending on the amount of fiber. Different void volumes, from 8% to 32% were obtained. When unmodified TMP was used for filaments manufacturing, BioPE1 produced filaments with higher void volume than BioPE2. 

The use of hydrophobized TMP fibers led to a general reduction of void volume. However, the reduction of void volume does not seem to depend on the used biopolymer (BioPE1 and BioPE2). Thus, the chemical compatibility between matrix and fibers and the MFI of BioPE are probably not the only parameters to consider in the production of filaments.

The lowest void fraction for both BioPE1 and BioPE2 matrices was achieved in the filaments complemented with 10% of LGT fibers, 7.73% and 10.21% respectively. Additionally, among the filaments complemented with 20% of fibers, B2-20LGT showed the lowest void fraction. These results are in accordance with the results of the filaments’ thickness variation. Therefore, the enzymatic grafting of LG onto TMP fibers’ surface could leads to the manufacturing of BioPE-based biocomposite filaments with a relative low porosity and limited thickness variation. This could presumptively improve the 3D printability. On the other hand, the filaments’ porosity was directly proportional to the fibers fraction in the filaments, since the higher the fibers load the higher the porosity.

### 3.2. SEM Analysis

**Figure 7:**
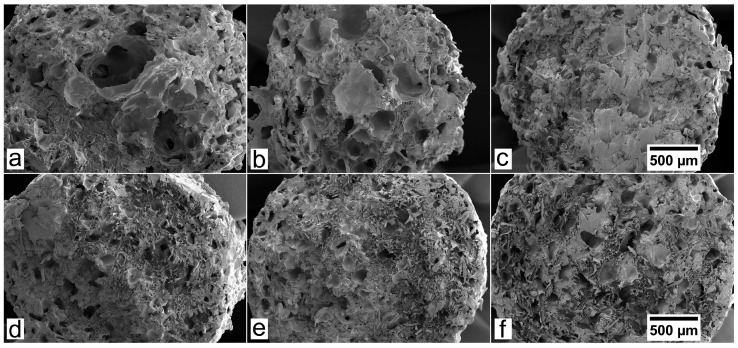
SEM images of the biocomposite filaments. BioPE1 containing 20% of untreated TMP fibers (**a**); 20% LG-treated TMP fibers (**b**); and 20% OG-treated TMP fibers (**c**). BioPE2 containing 20% of untreated TMP fibers (**d**); 20% LG-treated TMP fibers (**e**); and 20% OG-treated TMP fibers (**f**). All the images were acquired at 50× magnification in SEI mode.

The surface of filaments was analyzed by SEM in order to assess the chemical compatibility between the polymer and the TMP fibers, both unmodified and hydrophobized. Since the filaments complemented with 10% of fibers showed a relatively low void fraction, the main differences were found in the filaments with fiber loads of 20%. Such differences were notable for the BioPE1 series. As observed in Figure 7, the B1-20T filaments showed a high porosity, suggesting that the interfacial adhesion between the hydrophobic matrix and the hydrophilic TMP fibers was not satisfactory. However, the laccase-assisted grafting of LG onto TMP fibers enhanced the chemical compatibility with the BioPE1, since both B1-20LGT and B1-20OGT filaments showed a lower porosity than B1-20T. However, B1-20LGT filaments still exhibited a relatively high porosity, but lower than B1-20T. With respect to the BioPE2 series, the SEM pictures showed minor differences regarding the porosity of the filaments complemented with both unmodified and hydrophobized TMP fibers (Figure 7). Nevertheless, B2-20LGT filaments showed a homogeneous surface and a small fraction of micro-voids, suggesting that the laccase-assisted modification of TMP fibers with LG is a promising strategy for the manufacturing of low density filaments with a suitable roughness and porosity. Comparing both matrices, BioPE1-based filaments (Figure 7a–c) showed bigger voids and a notably higher porosity than BioPE2 series (Figure 7d–f). Therefore, the SEM images confirmed the trend observed in the measurement of the thickness variation and void fraction. It is worth mentioning that the low porosity of the BioPE2 series is expected to cause a lower water uptake and a better 3D printability, compared to the BioPE1 series.

### 3.3. Water Uptake

The water uptake of the filaments was measured to compare the two BioPE polymer matrices and also to assess the hydrophobic behavior of the enzymatically treated-TMP fibers. The water absorption of the biocomposites depends on several factors, mainly on the hygroscopic behavior of the fibers and the chemical compatibility between fibers and matrix, which affects the void volume and roughness. A good chemical compatibility of the matrix-fiber system could improve their interfacial adhesion, reducing the void fraction in the biocomposite. In addition, a high roughness of the filaments leads to a relatively high specific surface area, which will increase the contact between the filament and the water molecules. However, the chemical characteristics of fibers is expected to have an effect on water uptake, since the fiber hydrophobization act as a water repellent. Importantly, we demonstrated recently that the enzymatic treatment hydrophobized the TMP fibers and thus reduced the water uptake of PLA-based biocomposite filaments [22].

**Figure 8:**
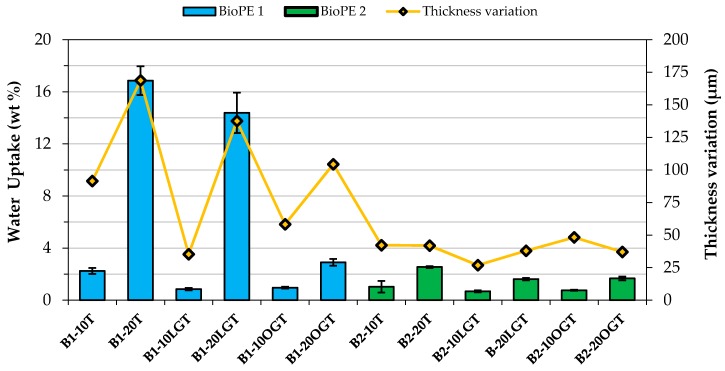
Water uptake (blue/green columns) of the biocomposite filaments after 32 days of water immersion (left axes). The thickness variation (yellow line) of the biocomposites is given in the right axis.

**Figure A1:**
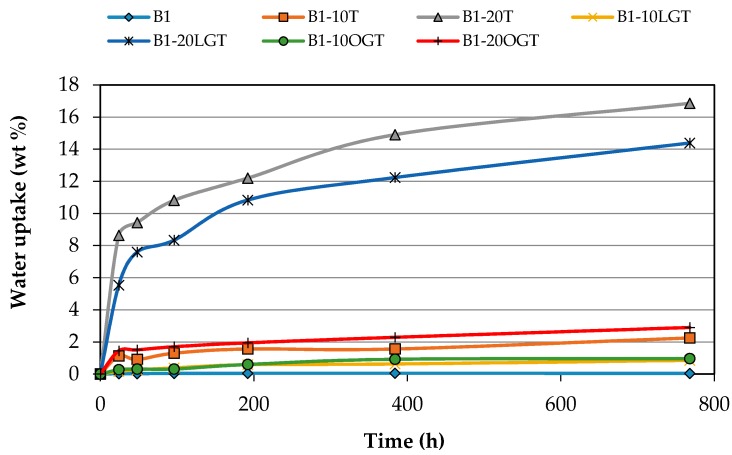
Water absorption (wt %) by the BioPE1-based filaments during 32 days of water immersion.

**Figure A2:**
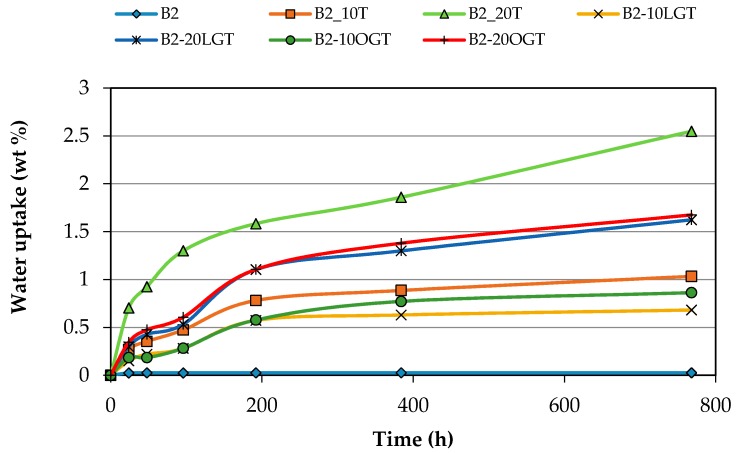
Water absorption (wt %) by the BioPE2-based filaments during 32 days of water immersion.

As observed in Table 2, the laccase-assisted grafting of the hydrophobic compound onto the TMP fibers reduced the void fraction of the biocomposites, with the exception of B2-20OGT. In addition, SEM images (Figure 7) confirmed that the interfacial adhesion between the matrix and the fibers was improved after the enzymatic treatment. Such effects had a clear impact on the water uptake of the filaments complemented with hydrophobized-TMP fibers (Figure 8, Figure A1 and Figure A2). For the BioPE1-based series, the enzymatic hydrophobization of the TMP fibers resulted in a major reduction of the filaments’ water uptake after 32 days. The water uptake has not completely flattened out after the 32 days (Figure A1 and Figure A2), but it clearly shows the different speed of water uptake of filaments based on BioPE1 and BioPE2. Such hydrophobic effects were especially significant for the filaments containing 20% fibers. For BioPE1 series, filaments complemented with 20% of LG-treated fibers reduced the water uptake, but to a lesser extent than B1-20OGT. Nonetheless, B1-20LGT series showed a much higher thickness variation than the B1-20OGT series, confirming the importance of manufacturing smooth and homogeneous filaments. The BioPE2-based filaments complemented with unmodified TMP fibers showed, on average, 30% higher water absorption than those in which the TMP fibers were previously hydrophobized by means of the laccase-mediated treatment. Generally, BioPE2-based biocomposite filaments absorbed lower amount of water than the BioPE1 series, especially for TMP fibers loads of 20%. As observed in Table 2 and Figure 7 the BioPE1 series complemented with fiber loads of 20% showed a much higher porosity than the BioPE2-based filaments, which affected clearly their water uptake behavior. Such results suggest that the MFI of the BioPE conditioned the manufacturing of the biocomposite filaments as well as their water uptake behavior.

### 3.4. 3D Printing

Polyethylene (PE) and more concretely HDPE are hardly ever used for 3D printing. In fact, as far as we know there is only one commercially available fully HDPE-based filament for 3D printing [37]. However, the manufacturer advises on their website that they do not have a reliable way to print such filament. Moreover, we could not find any scientific article focused on the manufacturing of PE-based biocomposite filaments for 3D printing. Generally, PE tends to shrink, bend, and warp when its temperature cools down, hindering remarkably the 3D printing process. Thus, the 3D printing of the manufactured BioPE-based biocomposite filaments is demanding.

The biocomposite filaments made from BioPE1 polymer were not suitable for 3D printing. The high MFI (20 g/10 min), due to its low viscosity, promoted warping and shrinkage problems during the 3D printing. Additionally, the relative high thickness variation of the BioPE1-based filaments hindered their feeding into the 3D printer. Nonetheless, BioPE2 biocomposite filaments showed a good 3D printing performance. As it was mentioned BioPE2 has a higher viscosity and a lower MFI (4.5 g/10 min) than BioPE1, which probably improved its 3D printability. Moreover, BioPE2-TMP biocomposites showed a better printability than the neat BioPE2. It is likely that the addition of TMP fibers reduced the MFI of the neat BioPE2 polymer [38], restricting the swelling and the shrinkage of the printed layers, and facilitating the corresponding shape fidelity and layers adhesion [39,40]. 

It is known that in fusion deposition modeling (FDM) amorphous polymers works better than crystalline polymers. Amorphous polymers have a disordered structure and a viscosity high enough to facilitate the adhesion of the layers and also maintain the shape of the printed layers [41]. On the contrary, highly crystalline polymers like HDPE develop partially crystalline structures upon cooling, resulting in distortions and internal stress [36]. Thus, highly crystalline polymers tend to shrink, hindering the shape fidelity of the printed layers and, therefore, limiting the 3D printing process [40]. Such a drawback could be solved, in part, with a heated print bed, which could reduce the cooling rate of the printed layers. However, for the 3D printing of big objects this inconvenience will probably appear again. Some interesting results were previously obtained by the Washington Open Object Fabricators (WOOF) team, who were able to 3D print a boat from recycled HDPE from milk jugs [42]. Nevertheless, they had to attach a heater to the extruder of the 3D printer in order to avoid the cooling down of the printed layers. In addition, they created a PE-based print bed since PE does not stick to any material other than PE. However, in this study and due to limitations of the printing unit, the 3D printing of BioPE2-based biocomposites filaments was performed without a heated print bed.

**Figure 9:**
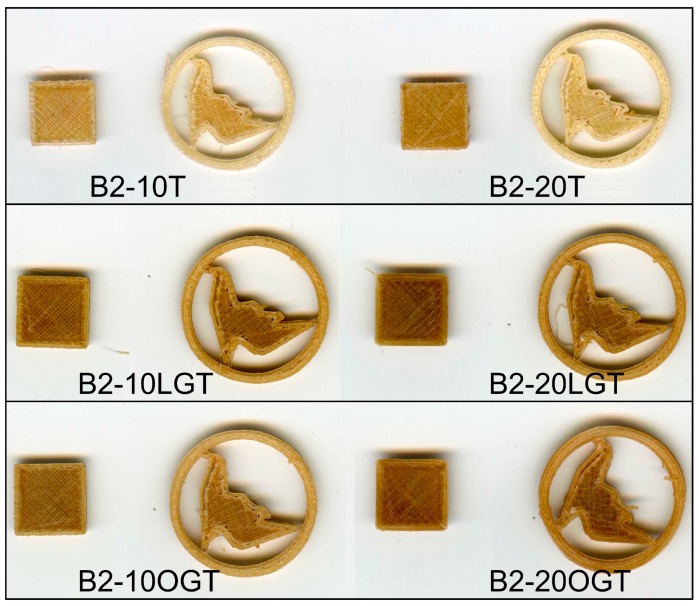
3D printed objects of the BioPE2 series, containing unmodified, LG, and OG-modified TMP fibers (10% and 20%). The squares are 10 mm × 10 mm. The circle is 20 mm in diameter.

Regarding the TMP fibers modification, objects printed with unmodified TMP showed a smoother surface for fibers loads of 10% than 20% fiber (Figure 9). Nonetheless, LG-TMP fibers showed a similar smoothness without warping or curling for both 10% and 20%, while OG-TMP fibers showed an improved quality in terms of smoothness at 20% fiber content. Thus, there was no major difference in quality between the 3D printed objects containing BioPE2 complemented with both modified and unmodified TMP fibers.

## 4. Conclusions

To the best of our knowledge, this is the first time that a scientific article focuses on the manufacturing of polyethylene-based biocomposite filaments for 3D printing. Two series of BioPE with different MFI were tested for the manufacturing of biocomposite filaments. BioPE1 with a relatively high MFI leads to the manufacturing of biocomposites with a high void fraction and high roughness. The high roughness had a notable impact on the water uptake behavior in some BioPE1 series. On the other hand, the relatively high MFI of the BioPE1 leads to warping and bending problems, and also to poor layer adhesion during 3D printing of BioPE1 biocomposite filaments. In addition, the relatively high porosity and thickness variation limited the 3D printing of BioPE1-based filaments.

The lower MFI of BioPE2 enabled the manufacturing of biocomposite filaments suitable for 3D printing. Moreover, the 3D printing of BioPE2 was improved with the addition of TMP fibers. The hydrophobicity of the fibers was tailored by means of laccase-assisted grafting of OG or LG compounds. Hence, filaments complemented with enzymatically treated TMP fibers showed a remarkably lower water uptake compared with those filled with unmodified TMP fibers. No major differences were observed with respect to the 3D print quality and water uptake behavior of the filaments containing OG and LG-treated TMP fibers. Finally, it is worth mentioning that the biocomposites developed in this study may be plausible materials for injection molding operations and products where low water uptake is required.

## Figures and Tables

**Table 1:** Composition of biocomposite filaments for 3D printing.

Code	BioPE	BioPE (Weight %)	TMP fiber (Weight %)	TMP fiber modification
B1	BioPE1	100	-	-
B1-10T	BioPE1	90	10	-
B1-20T	BioPE1	80	20	-
B1-10LGT	BioPE1	90	10	LG
B1-20LGT	BioPE1	80	20	LG
B1-10OGT	BioPE1	90	10	OG
B1-20OGT	BioPE1	80	20	OG
B2	BioPE2	100	-	-
B2-10T	BioPE2	90	10	-
B2-20T	BioPE2	80	20	-
B2-10LGT	BioPE2	90	10	LG
B2-20LGT	BioPE2	80	20	LG
B2-10OGT	BioPE2	90	10	OG
B2-20OGT	BioPE2	80	20	OG

**Table 2:** Measurement of the % of void fraction in the manufactured filaments.

Sample	Theoretical density (g/cm^3^)	Measured density, Equations (1) and (2) (g/cm^3^)	Void fraction (vol %)
B1	0.955	0.9538	0.1
B1-10T	0.8595	0.6761	27.1
B1-20T	0.764	0.5199	47.0
B1-10LGT	0.8595	0.7930	8.4
B1-20LGT	0.764	0.5922	29.0
B1-10OGT	0.8595	0.7746	11.0
B1-20OGT	0.764	0.6466	18.2
B2	0.954	0.953	0.1
B2-10T	0.8586	0.697	23.2
B2-20T	0.763	0.6143	24.2
B2-10LGT	0.8586	0.7709	11.4
B2-20LGT	0.763	0.6741	13.2
B2-10OGT	0.8586	0.739	16.2
B2-20OGT	0.763	0.5994	27.3
